# Pain perception in turkish adolescents with fmf and their mothers: a preliminary report

**DOI:** 10.1186/1546-0096-12-S1-P103

**Published:** 2014-09-17

**Authors:** Deniz Bayraktar, Nursen Ilcin, Ozge Altug Gucenmez, Sema Savci, Balahan Makay, Erbil Unsal

**Affiliations:** 1 School of Physical Therapy and Rehabilitation, Dokuz Eylul University, Izmir, Turkey; 2 Pediatric Rheumatology, Dokuz Eylul University, Izmir, Turkey

## Introduction

Familial Mediterranean fever (FMF) is the most common inherited autoinflammatory disease in the world and the patients suffer from recurrent self-limiting episodes of fever and painful polyserositis, particularly peritonitis, pleuritis and arthritis.

## Objectives

To determine whether there is a difference in pain perception between adolescents with FMF and their mothers’.

## Methods

Adolescents age 13-18 with a certain diagnosis of FMF and their mothers were invited to participate in the study. Demographic and clinical characteristics of the patients were obtained from hospital records and family interview. Pain perception was measured in rest and in activity with Visual Analog Scale 0-100 mm form.

## Results

Fourteen children (10 Female) and their mothers were included in the study so far. The mean age was 15.21±1.37 years (min-max: 13-17 years), the mean duration of disease was 78.32±66.00 months (max-min: 2.5-180 months), the mean time since diagnosis was 53.36±63.40 months (min-max: 1-174 months). Mothers’ mean age was 39.93±5.38 years. The mean pain perception in rest was evaluated as 35.00±27.69 mm and 46.64±35.26 mm in adolescents and mothers, respectively (p=0.34). The mean pain perception in activity was determined as 63.29±32.11 mm and 67.93±31.86 mm (p=0.70) in adolescents and mothers, respectively.

## Conclusion

According to our primary findings even though mothers have slightly greater pain perception for their children there is no statistically significant difference in pain perception between Turkish adolescents with FMF and their mothers. However, it should be kept in mind these are the preliminary results. Further studies including fathers and other relatives needed make permanent decision about pain perception of different family members.

## Disclosure of interest

None declared.

